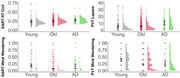# Sensitivity of Brief Sustained Attention Measures as Markers for Very Mild Alzheimer's Disease

**DOI:** 10.1002/alz70857_101639

**Published:** 2025-12-25

**Authors:** Matthew S. Welhaf, Andrew J. Aschenbrenner, John C. Morris, Jason J. Hassenstab

**Affiliations:** ^1^ Washington University in St. Louis, St. Louis, MO, USA; ^2^ Knight Alzheimer Disease Research Center, St. Louis, MO, USA

## Abstract

**Background:**

Attentional deficits have been identified as an early marker of cognitive change in Alzheimer's disease (AD). However, studies typically focus on response inhibition tasks (e.g., Stroop). Sustained attention, or our ability to maintain attention over time, is important for everyday activities including driving and is understudied in AD. Processes contributing to such AD‐related differences might reflect either executive control failures or lower levels of arousal, although this remains unclear. We addressed this issue by measuring both executive‐ and arousal‐based sustained attention in two sustained attention tasks.

**Method:**

Five‐hundred and nine adults were classified as Young Adults (*N* = 115; Aged 18‐35) Cognitively Normal (CN) Older Adults (*N* = 354; Aged 65–93; Clinical Dementia Rating® (CDR) = 0) and Older Adults with very mild AD (*N* = 40; Aged 65–91, CDR=0.5). All completed brief (< 5 minute) versions of the Sustained Attention to Response Task (SART), measuring executive‐based sustained attention, and the Psychomotor Vigilance Task (PVT), measuring arousal‐based sustained attention. Sustained attention was assessed using SART response time (RT) variability and PVT lapses (RTs > 500 ms) and mind wandering rates in each task.

**Results:**

Significant group differences were evident in both the SART (*p* = .013) and the PVT (*p* < .001; see Figure 1). Older adults with very mild AD performed worse on both the SART and PVT compared to younger adults (*p*'s < .05). However, performance differences between CN older adults and those with very mild AD were only evident in the PVT (*p* < .001). CN older adults reported less mind wandering than younger adults (*p*'s < .001). However, older adults with very mild AD reported more mind wandering that CN older adults in the SART (*p* = .018).

**Conclusion:**

Individuals in the very earliest stages of AD appear to show performance deficits in the arousal‐based sustained attention. However, in terms of mind wandering, individuals in the very earliest stages of AD had more mind wandering during the executive‐based SART. Collectively, sustained attention deficits in very early AD arise in both arousal and executive components but might manifest differently depending on how sustained attention ability is measured.